# Association between hemoglobin-to-red blood cell distribution width ratio and cognitive impairment in elderly Americans

**DOI:** 10.1097/MD.0000000000042365

**Published:** 2025-05-09

**Authors:** Minggeng Jiang, Shicong Hong, Yingying Li, Xiaoxiao Zhu

**Affiliations:** a The Third People’s Hospital of Liaocheng City, Liaocheng, Shandong Province, China.

**Keywords:** cognitive impairment, hemoglobin, hemoglobin-to-red blood cell distribution width ratio, old adults, red blood cell distribution width ratio.

## Abstract

This study aimed to investigate the potential association between the hemoglobin-to-red blood cell distribution width ratio (HRR) and cognitive impairment in a representative sample of elderly Americans. Data from National Health and Nutrition Examination Survey 1999 to 2002 were utilized to analyze demographic information, cognitive function assessments, and laboratory data. Binary Logistic regression was conducted to analyze the associations. Subgroup analyses and sensitivity analyses were performed simultaneously. In the univariable logistic regression analysis, the odds ratio was 0.13 (95% confidence interval [CI]: 0.06–0.25; *P* < .001). After adjusting for all covariates in the multivariable regression analysis, the odds ratio was 0.25 (95% CI: 0.11–0.6; *P* = .002). Compared to the lowest HRR group, the higher HRR groups had significantly lower odds of cognitive impairment: Q2 (OR = 0.6, 95% CI: 0.42–0.84, *P* = .003), Q3 (OR = 0.54, 95% CI: 0.37–0.77, *P* = .001), and Q4 (OR = 0.56, 95% CI: 0.38–0.82, *P* = .003). Subgroup and sensitivity results were stable and consistent. These results suggest that higher levels of HRR may be associated with a lower risk of cognitive impairment in elderly Americans.

## 1. Introduction

Cognitive impairment refers to difficulties with memory, learning, concentration, executive functioning, and other cognitive abilities. In its severe stage, it is known as dementia and primarily affects older individuals. Cognitive impairment and dementia impact tens of millions of people globally, causing significant distress to patients and caregivers, as well as imposing a financial burden on families and healthcare systems.^[1–4]^ The worldwide elderly population is steadily increasing, leading to a rapid growth in the number of individuals affected by cognitive impairment. It is projected that by 2050, the number of people affected by dementia will reach a staggering 152 million.^[5]^ Diagnosing cognitive impairment relies mainly on cognitive assessments, blood tests, cerebrospinal fluid biomarkers, cranial MRI, PET scans, and other methods. Determining the etiology of cognitive impairment is challenging, as there is no simple or laboratory test to identify the specific type of cognitive impairment. Research suggests that increased pro-inflammatory factors and neuronal damage caused by inflammation may be the cause of cognitive impairment.^[6,7]^ Hemoglobin-to-red blood cell distribution width ratio (HRR), calculated by dividing hemoglobin (Hb) by red blood cell distribution width ratio (RDW), is a novel marker of inflammation proposed by Sun et al.^[8]^ Previous investigations have shown that HRR was associated with inflammation.^[9]^

However, the association between HRR and cognitive dysfunction is unclear. and in this study, we aimed to test the potential association between HRR and cognitive impairment in elderly Americans.

## 2. Materials and methods

### 2.1. Data source

This study used data from the National Health and Nutrition Examination Survey (NHANES) 1999 to 2002 for analysis. The NHANES program began in the early 1960s and is conducted as a series of surveys focusing on various health topics. The sample for the NHANES survey was selected to represent the noninstitutionalized population of the United States. More information about the background, design, and operation of NHANES is available on the NHANES web site (https://www.cdc.gov/ nchs/nhanes/about_nhanes.htm).

### 2.2. Ethics statement

All NHANES data were de-identified, and all participants provided written informed consent consistent with National Center for Health Statistics Institutional Review Board approval. Institutional Review Board approval and participant signed informed consent were waived for NHANES data analysis.

### 2.3. Study subjects

**Figure 1.**
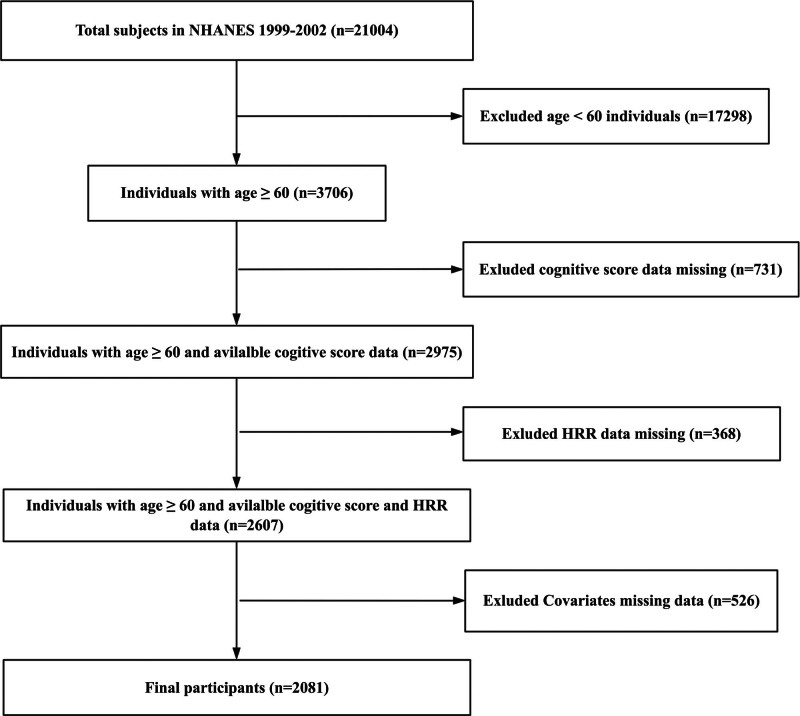
Flowchart of participant selection from the National Health and Nutrition Examination Survey (NHANES) 1999 to 2002. *Description*: Diagram illustrates the exclusion cascade from initial 21,004 NHANES participants to final analytic cohort (n = 2081) with complete cognitive and hematologic data. Exclusions: age < 60 years (n = 17,298), missing cognitive testing (n = 731), missing hemoglobin/RDW data (n = 368), missing covariates (n = 526).

Participants aged 60 years or older underwent the digit symbol substitution test (DSST) to assess cognitive function, and complete blood count data were included in the present study.^[10]^ Subjects with incomplete DSST assessments or without covariates data were excluded. In the NHANES database, a total of 21,004 people were included in the screening from 1999 to 2002, of which 17,298 people younger than 60 years old were excluded, leaving 3706 people, 731 people with missing cognitive tests were excluded, leaving 2975 people, and 368 people with missing HRR data were excluded. There were 2607 people left, 526 people with missing covariates were excluded, and 2081 people were finally included in this study. Figure 1 shows the selection process.

### 2.4. Cognitive impairment

Cognitive impairment was defined as a DSST score below the lowest quartile. The DSST requires the subject to accurately code a series of symbols within 120 seconds. This task requires reaction speed, sustained attention, visuospatial skills, associative learning and memory and is considered a sensitive measure of cognitive function.^[10]^ The DSST is a paper-and-pencil test of psychomotor performance in which subjects are given a key grid of numbers and matching symbols, and a test section with numbers and empty boxes. The test consists of filling as many empty boxes as possible with symbols that match each number. The score is the number of correct number-symbol matches achieved within 90 seconds.

### 2.5. Hemoglobin-to-red blood cell distribution width ratio

Using laboratory data from NHANES, hemoglobin-to-red blood cell distribution width ratio (HRR) calculated by dividing hemoglobin (Hb) by red blood cell distribution width ratio (RDW).^[9,11]^

### 2.6. Covariates

To reduce the possible confounding effects of HRR on cognition, we controlled for several variables: age, gender, race, education, poverty income ratio (PIR), body mass index (BMI), smoking, alcohol drinking, diabetes, coronary heart disease, stroke, hypertension. In NHANES, age is coded up to 85 years to protect participant confidentiality, and cognitive function is clearly related to age, so age groupings were constructed to reduce the impact of many 85-year-olds. Ages are divided into 3 groups: 60 to <70 years, 70 to <80 years, and ≥80 years; Race/ethnicity is classified as Mexican American, other Hispanic, non-Hispanic White or non-Hispanic Black, and other races; Education level is classified as less than high school, high school graduate, or college or higher; PIR is classified as: low-income: PIR < 1.3, middle-income: PIR 1.3 to 3.5, high-income: PIR ≥ 3.5; BMI is a continuous variable and is weight (kg)/height (m)^[2]^; Smoking is determined by the NHANES questionnaire, which is defined as smoking at least 100 cigarettes in a lifetime, alcohol consumption is also determined by the NHANES questionnaire, which is defined as drinking at least 12 alcoholic beverages per 1 year, and if the participant self-reports being told by a physician that he or she has diabetes, coronary heart disease, stroke, hypertension is a history.

### 2.7. Statistical analysis

We conducted a descriptive analysis of all participants. Categorical data were presented as numbers (percentages), while continuous data were reported as either the mean ± standard deviation or median (interquartile range) as appropriate. The differences in categorical variables between the 2 groups were assessed using the Chi-square test or Fisher exact test as appropriate. For continuous variables, the Student *t* test or rank-sum test was utilized to compare between the 2 groups. Multivariable logistic regression analyses (odds ratios [OR], confidence interval [CI]) were performed to evaluate the independent association between HRR and Cognitive Impairment. Multifactor analysis made 3 models, Model 1 adjusted for age, sex, race, education, pir. Model 2 was additionally adjusted for BMI, smoking, alcohol drinking. Model 3 additionally adjusted for diabetes, coronary heart disease, stroke, hypertension. Subgroup analysis was also conducted, and the grouping variables were: age, gender, education level, PIR, alcohol consumption, diabetes, stroke, and hypertension. Sensitivity analysis used multiple imputation to impute missing covariates, followed by multivariable analysis. All analyses were conducted using the statistical software packages R (http://www.R-project.org, The R Foundation) and Free Statistics software version 1.9.2. A 2-tailed test was applied, and a *P* < .05 was considered statistically significant in our study.

## 3. Results

### 3.1. Demographic characteristics

A total of 2081 adults aged 60 years or older participated in the 1999 to 2000 and 2001 to 2002 NHANES cycles and were included in the final analysis, including 1033 males and 1048 females. Among them, 508 individuals (24.4%) had cognitive impairment. The cognitive impairment group differed significantly from the noncognitive impairment group in terms of gender (*P* = .011), age (*P* < .001), race (*P* < .001), education level (*P* < .001), PIR (*P* < .001), alcohol consumption (*P* < .001), diabetes (*P* < .001), stroke (*P* < .001), hypertension (*P* = .017), and HRR (*P* < .001).However, there were no statistically significant differences between the 2 groups in terms of smoking status (*P* = .592), coronary heart disease (*P* = .36), and BMI (*P* = .721) (see Table 1).

**Table 1:** Baseline characteristics of study participants stratified by cognitive status.

	Total (n = 2081)	Noncognitive impairment (n = 1573)	Cognitive impairment (n = 508)	*P*
Gender, n (%)				
Male	1033 (49.6)	756 (48.1)	277 (54.5)	.011
Female	1048 (50.4)	817 (51.9)	231 (45.5)	
Age, n (%)				
60 to 69	966 (46.4)	778 (49.5)	188 (37.0)	<.001
70 to 79	692 (33.3)	525 (33.4)	167 (32.9)	
≥80	423 (20.3)	270 (17.2)	153 (30.1)	
Race, n (%)				
Mexican American	385 (18.5)	215 (13.7)	170 (33.5)	<.001
Other Hispanic	77 (3.7)	43 (2.7)	34 (6.7)	
Non-Hispanic White	1288 (61.9)	1106 (70.3)	182 (35.8)	
Non-Hispanic Black	289 (13.9)	176 (11.2)	113 (22.2)	
Other Race	42 (2.0)	33 (2.1)	9 (1.8)	
Education, n (%)				
<High school	798 (38.3)	415 (26.4)	383 (75.4)	<.001
High school	512 (24.6)	436 (27.7)	76 (15)	
>High school	771 (37.0)	722 (45.9)	49 (9.6)	
PIR, n (%)				
Low-income (PIR < 1.3)	562 (27.0)	299 (19)	263 (51.8)	<.001
Middle-income (PIR 1.3–3.5)	907 (43.6)	702 (44.6)	205 (40.4)	
High-income (PIR ≥ 3.5)	612 (29.4)	572 (36.4)	40 (7.9)	
Smoking, n (%)				
No	974 (46.8)	731 (46.5)	243 (47.8)	.592
Yes	1107 (53.2)	842 (53.5)	265 (52.2)	
Alcohol drinking, n (%)				
No	800 (38.4)	572 (36.4)	228 (44.9)	<.001
Yes	1281 (61.6)	1001 (63.6)	280 (55.1)	
Diabetes, n (%)				
NO	1720 (82.7)	1347 (85.6)	373 (73.4)	<.001
YES	361 (17.3)	226 (14.4)	135 (26.6)	
Coronary heart disease, n (%)				
No	1879 (90.3)	1415 (90)	464 (91.3)	.36
Yes	202 (9.7)	158 (10)	44 (8.7)	
Stroke, n (%)				
No	1944 (93.4)	1495 (95)	449 (88.4)	<.001
Yes	137 (6.6)	78 (5)	59 (11.6)	
Hypertension, n (%)				
No	1001 (48.1)	780 (49.6)	221 (43.5)	.017
Yes	1080 (51.9)	793 (50.4)	287 (56.5)	
BMI, Mean ± SD	27.5 ± 5.1	27.5 ± 5.1	27.4 ± 5.2	.721
HRR, Mean ± SD	1.1 ± 0.1	1.1 ± 0.1	1.1 ± 0.2	<.001

BMI = body mass index, HRR = hemoglobin-to-red blood cell distribution width ratio, PIR = poverty income ratio.

### 3.2. Logistic regression analyses

In the analysis of the association between HRR and cognitive impairment, our results revealed a significant negative association. In the univariate logistic regression analysis, the odds ratio was 0.13 (95% CI: 0.06–0.25; *P* < .001). In univariate analysis, the study found that diabetes, age, female, gender, education level, PIR, alcohol consumption, stroke, hypertension and cognitive impairment were statistically different. Specifically, female gender, education level, PIR, and alcohol consumption showed protective factors; while diabetes, age, stroke, and hypertension were risk factors. Additionally, no statistical differences were observed between smoking, BMI, coronary heart disease, and cognitive impairment. After adjusting for all covariates in the multivariable regression analysis, HRR as a continuous variable， the odds ratio was 0.25 (95% CI: 0.11–0.6; *P* = .002). HRR as a categorical variable，We further divided HRR into quartiles and conducted multivariable regression analyses using 3 other models, Compared to the lowest HRR group (Q1, range: 0.308–1.023), the adjusted OR for cognitive impairment in the higher HRR groups were as follows: Q2 (1.023–1.119) had an odds ratio of 0.6 (95% CI: 0.42–0.84; *P* = .003), Q3 (1.119–1.205) had an odds ratio of 0.54 (95% CI: 0.37–0.77; *P* = .001), and Q4 (1.205–1.476) had an odds ratio of 0.56 (95% CI: 0.38–0.82; *P* = .003) (see Table 2).

**Table 2:** Multivariable logistic regression analyses of hemoglobin-to-red cell distribution width ratio and cognitive impairment.

	Cognitive impairment/n	Prevalence/%	Non-adjusted mode		Model 1		Model 2		Model 3	
			OR (95% CI)	*P*	OR (95% CI)	*P*	OR (95% CI)	*P*	OR (95% CI)	*P*
HRR (continuous)	508/2081	24.4	0.13 (0.06–0.25)	<.001	0.23 (0.1–0.54)	.001	0.24 (0.1–0.55)	.001	0.25 (0.11–0.6)	.002
HRR categories										
Quantile 1	180/520	34.6	1 (Ref)		1 (Ref)		1 (Ref)		1 (Ref)	
Quantile 2	113/515	21.9	0.53 (0.4–0.7)	<.001	0.6 (0.43–0.84)	.003	0.6 (0.43–0.84)	.003	0.6 (0.42–0.84)	.003
Quantile 3	109/523	20.8	0.5 (0.38–0.66)	<.001	0.52 (0.37–0.75)	<.001	0.52 (0.36–0.74)	<.001	0.54 (0.37–0.77)	.001
Quantile 4	106/523	20.3	0.48 (0.36–0.63)	<.001	0.54 (0.37–0.78)	.001	0.54 (0.38–0.79)	.001	0.56 (0.38–0.82)	.003
*P* for trend				<.001	.001		.001		.002	

Model 1 adjusted for age, sex, race, education, PIR. Model 2 was additionally adjusted for BMI, smoking, alcohol drinking. Model 3 additionally adjusted for Diabetes, Coronary heart disease, Stroke, Hypertension.

BMI = body mass index, HRR = hemoglobin-to-red blood cell distribution width ratio, PIR = poverty income ratio.

### 3.3. Subgroup analyses

**Figure 2.**
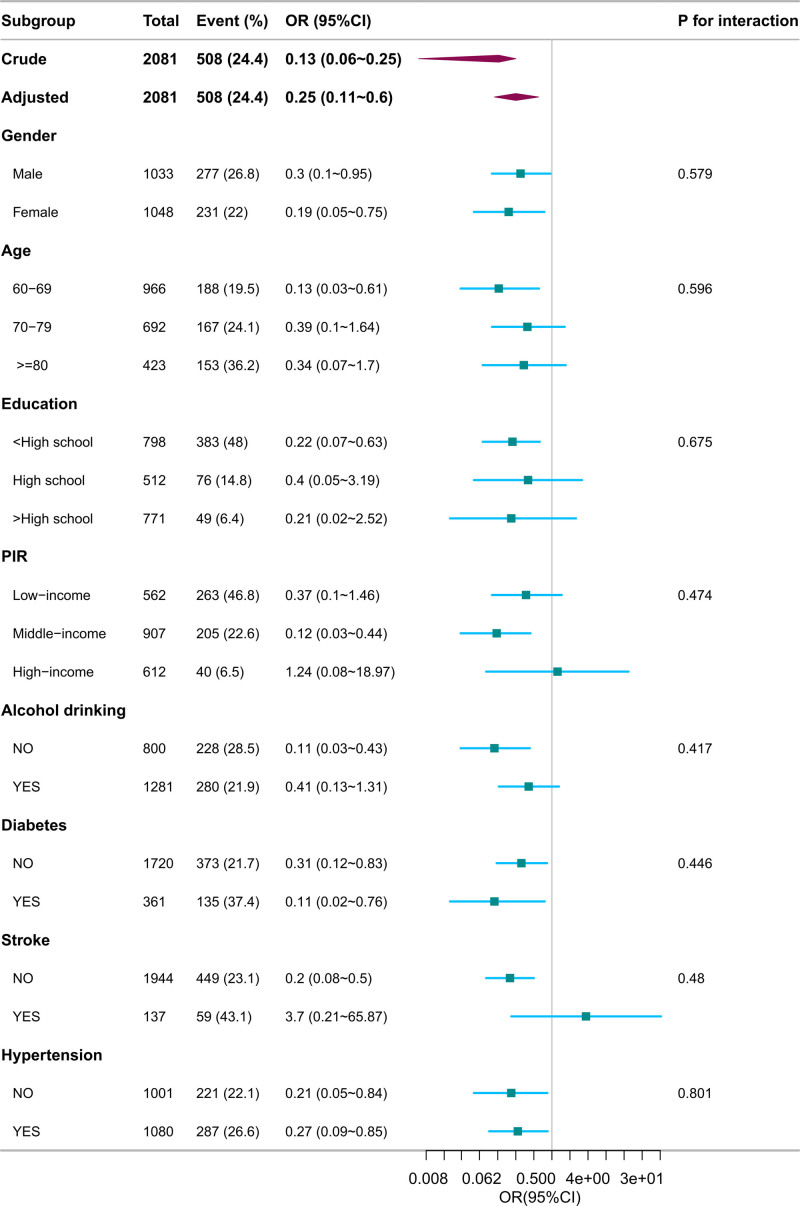
Subgroup analysis of the association between hemoglobin-to-red blood cell distribution width ratio (HRR) and cognitive impairment. *Description*: Forest plot displays stratified odds ratios (ORs) and 95% confidence intervals (CIs) by age, gender, education, and comorbidities. Interaction *P*-values > .05 indicate consistent associations across subgroups.

In order to verify the stability of the results, we conducted subgroup analyses. The study conducted subgroup analyses using stratified regression models based on gender, age, education level, PIR, alcohol consumption, diabetes, stroke, and hypertension. The results indicated that there was no statistically significant interaction between hemoglobin-red cell distribution width ratio and the aforementioned factors with cognitive impairment (all *P* for interaction were > .05), as shown in Figure 2.

### 3.4. Sensitivity analyses

We also performed some sensitivity analyses. first, We reanalyzed the data after multiple imputation of the missing 526 covariates, and performed a multivariable logistic regression analysis to ensure the stability of our study results. The results still showed a negative correlation between HRR and cognitive impairment. Additionally, we conducted multivariable regression analyses using 3 other models to demonstrate the stability of the results, which also indicated a negative correlation between HRR and cognitive impairment. We further divided HRR into quartiles and conducted multivariable regression analyses using 3 other models, which still showed a negative correlation between HRR and cognitive impairment (see Supplementary Material S1, Supplemental Digital Content, https://links.lww.com/MD/O844). Furthermore, we conducted stratified and interaction analyses to explore whether the negative correlation between HRR and cognitive impairment is influenced by gender, age, education level, PIR, alcohol consumption, diabetes, stroke, and hypertension (see Supplementary Material S2, Supplemental Digital Content, https://links.lww.com/MD/O845). The interaction analysis showed that none of the aforementioned factors significantly interfered with the negative correlation between HRR and cognitive impairment (all *P* for interaction > .05).

## 4. Discussion

In this retrospective cross-sectional study, we investigated the relationship between the hemoglobin-to-red blood cell distribution width ratio (HRR) and cognitive impairment and identified significant associations. After adjusting for all covariates, the risk of cognitive impairment was reduced by 75%. We demonstrate that HRR is inversely associated with cognitive impairment after adjusting for possible confounders. Simultaneous subgroup and sensitivity analysis results were similar. This study highlights the importance of HRR (hemoglobin-to-red blood cell distribution width ratio) in cognitive function. HRR is a metric easily obtained from routine laboratory databases without additional technology or expense and serves as an indicator of a patient’s level of inflammatory response.^[8,12–16]^ Notably, although HRR itself is not directly related to cognitive dysfunction, its correlation with the degree of inflammation suggests a potential link between systemic inflammation and cognitive impairment in the brain.^[17]^

In recent studies, low HRR levels have emerged as a predictor of poor prognosis in various critical illness conditions, including coronary heart disease, sepsis, and ischemic stroke.^[18–20]^ These findings suggest that HRR may reflect a broader inflammatory state that may have implications for cognitive health. Of particular interest is the relationship between hemoglobin levels and cognitive performance. High hemoglobin levels were non-significantly associated with reduced cognitive performance, indicating a nonlinear relationship between these 2 variables.^[21]^ Another study showed that low hemoglobin levels in the blood are associated with poor cognitive function and AD.^[22]^ Furthermore, anemia is associated with a significantly increased risk of all-cause dementia, and hemoglobin (HGB) and red blood cell distribution width (RDW) are causally associated with Alzheimer disease risk.^[23,24]^ These associations highlight the importance of considering hematological measures in the context of cognitive health.

In our study, HRR is hypothesized to be a relevant marker for assessing the risk of cognitive impairment. Since HRR is calculated based on HGB and RDW, its decrease may be attributed to low HGB levels, high RDW values, or both. This finding is consistent with previous studies showing that low HGB and high RDW are associated with increased risk of cognitive decline. Specifically, Decreased HGB or/and increased RDW are positively associated with cognitive impairment, further strengthening the role of HRR in predicting cognitive dysfunction.

Additionally, elevated RDW levels within the normal range may indicate insufficient hematopoiesis or destruction of red blood cells, which may lead to a chronic inflammatory state. In fact, higher RDW levels are associated with an underlying inflammatory state and poor prognosis.^[25–27]^ These findings suggest that, as a composite marker combining HGB and RDW, HRR may provide a more comprehensive assessment of the impact of the inflammatory environment on cognitive health.

Taken together, our findings suggest that, as a simple and informative laboratory measure, HRR has the potential to be a valuable tool for predicting and managing cognitive impairment. Future studies should explore the mechanistic links between inflammation, hematological markers, and cognitive decline to further elucidate the role of HRR in this complex interaction.

However, our study also has some limitations. First, since our study adopted a retrospective cross-sectional design, we were unable to determine the causal relationship between HRR and cognitive dysfunction. Second, our sample size was small and only involved older adults in the United States, so our results may not be generalizable to the entire population. Finally, our study did not consider other potential biomarkers, which may affect our understanding of the relationship between HRR and cognitive dysfunction.

In view of the above findings and limitations, future larger-scale and long-term studies are needed to verify our conclusions and further explore the potential role of HRR in cognitive impairment.

## 5. Conclusions

In conclusion, this study provides robust evidence that HRR is an independent risk factor for cognitive impairment in older adults in the United States. Results showed that HRR had a significant effect on cognitive impairment after adjusting for covariates. As HRR increases, the risk of cognitive impairment decreases, and the 2 are inversely related.

## Author contributions

**Conceptualization:** Minggeng Jiang, Shicong Hong.

**Data curation:** Shicong Hong.

**Formal analysis:** Minggeng Jiang.

**Investigation:** Minggeng Jiang.

**Methodology:** Minggeng Jiang.

**Project administration:** Xiaoxiao Zhu.

**Resources:** Minggeng Jiang.

**Software:** Yingying Li.

**Supervision:** Xiaoxiao Zhu.

**Validation:** Shicong Hong, Yingying Li.

**Visualization:** Yingying Li.

**Writing – original draft:** Minggeng Jiang.

**Writing – review & editing:** Yingying Li.

## Supplementary Material


