# Supplementary material for: Association between hemoglobin-to-red blood cell distribution width ratio and cognitive impairment in elderly Americans
Source: Medicine (Baltimore). 2025 May 9;104(19):e42365. doi: 10.1097/MD.0000000000042365 (PMC12073934; doi:10.1097/MD.0000000000042365)
Supplement: Supplementary file 1 [file medi-104-e42365-s001.pdf]

Supplemental Digital Content S1. Multiple Imputation Analysis of Hemoglobin-to-Red Cell Distribution Width Ratio and Cognitive Impairment

	Cognitive impairment/n	Prevalence/%	Non-adjusted Mode		Model 1		Model 2		Model 3	
			OR (95% CI)	*P*	OR (95% CI)	*P*	OR (95% CI)	*P*	OR (95% CI)	*P*
HRR(continuous)	632/2607	24.2	0.16 (0.09~0.29)	<0.001	0.35 (0.16~0.74)	0.006	0.35 (0.17~0.76)	0.007	0.39 (0.18~0.84)	0.016
HRR categories										
Quantile 1	221/652	33.9	1(Ref)		1(Ref)		1(Ref)		1(Ref)	
Quantile 2	139/649	21.4	0.53 (0.41~0.68)	<0.001	0.64 (0.47~0.86)	0.003	0.64 (0.47~0.86)	0.003	0.65 (0.48~0.87)	0.005
Quantile 3	141/654	21.6	0.54 (0.42~0.69)	<0.001	0.66 (0.48~0.9)	0.008	0.66 (0.48~0.9)	0.009	0.69 (0.5~0.95)	0.022
Quantile 4	131/652	20.1	0.49 (0.38~0.63)	<0.001	0.62 (0.45~0.86)	0.005	0.63 (0.45~0.88)	0.006	0.65 (0.46~0.9)	0.011
P for trend				<0.001	0.006		0.007		0.015	

Model1 adjusted for age, sex, race, education, pir. Model2 was additionally adjusted for BMI, smoking, alcohol drinking. Model 3 additionally adjusted for Diabetes, Coronary heart disease, Stroke, Hypertension.
