# Supplementary material for: Association between hemoglobin-to-red blood cell distribution width ratio and cognitive impairment in elderly Americans
Source: Medicine (Baltimore). 2025 May 9;104(19):e42365. doi: 10.1097/MD.0000000000042365 (PMC12073934; doi:10.1097/MD.0000000000042365)

Supplemental Digital Content S2. Sensitivity and subgroup analyses of HRR-cognitive impairment association.

Description: Forest plot presents stratified associations between hemoglobin-to-red cell distribution width ratio and cognitive impairment across subgroups of age (60-69/70-79/≥80 years), gender (male/female), education level (<high school/high school/>high school), poverty-income ratio (<1.3/1.3-3.5/≥3.5), alcohol consumption (yes/no), diabetes (yes/no), stroke (yes/no), and hypertension (yes/no), with all interaction p-values >0.05 indicating consistent effects (multiply imputed data, n=2,607).

Subgroup	Total	Event (%)	OR (95%CI)	P for interaction
**Crude**	**2607**	**632 (24.2)**	**0.16 (0.09~0.29)**	
**Adjusted**	**2607**	**633 (24.2)**	**0.39 (0.18~0.84)**	
**Age**				
60~69	1216	238 (19.6)	0.35 (0.09~1.34)	0.415
70~79	857	215 (25.1)	0.32 (0.09~1.18)	
>=80	534	179 (33.5)	0.66 (0.15~2.9)	
**Gender**				
Male	1280	330 (25.8)	0.44 (0.16~1.26)	0.657
Female	1327	302 (22.8)	0.34 (0.1~1.14)	
**Education**				
<High school	1052	488 (46.4)	0.36 (0.14~0.91)	0.583
High school	623	86 (13.8)	0.35 (0.05~2.19)	
>High school	932	58 (6.2)	0.5 (0.05~4.98)	
**PIR**				
Low−income	729	335 (46)	0.81 (0.24~2.74)	0.098
Middle−income	1140	245 (21.5)	0.14 (0.04~0.46)	
High−income	738	52 (7)	0.54 (0.05~5.67)	
**Diabetes**				
NO	2155	478 (22.2)	0.39 (0.16~0.95)	0.982
Yes	452	154 (34.1)	0.36 (0.07~1.93)	
**Stroke**				
NO	2437	561 (23)	0.34 (0.15~0.77)	0.722
Yes	170	71 (41.8)	1.95 (0.15~24.77)	
**Hypertension**				
NO	1260	284 (22.5)	0.34 (0.1~1.12)	0.829
Yes	1347	348 (25.8)	0.4 (0.14~1.13)	
**Alcohol drinking**				
NO	1032	293 (28.4)	0.21 (0.06~0.69)	0.532
Yes	1575	339 (21.5)	0.63 (0.22~1.8)	

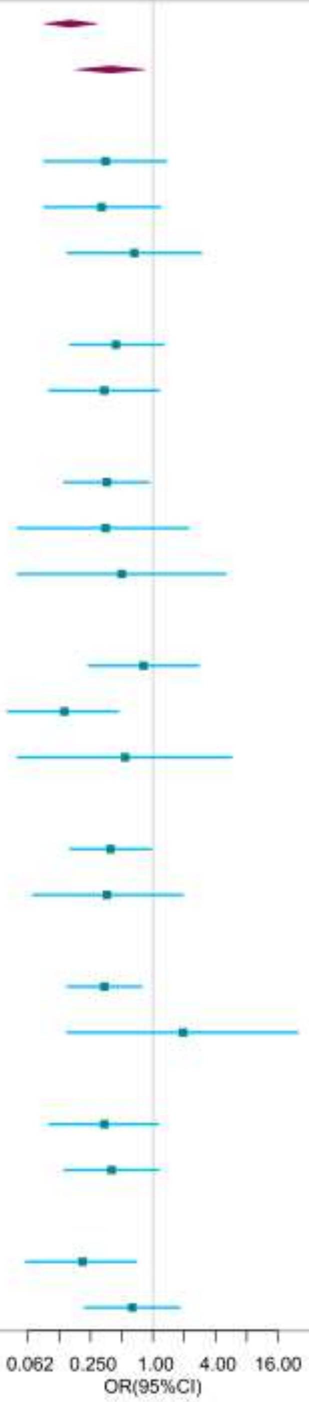

Supplement: Supplementary file 2 [file medi-104-e42365-s002.pdf]
